# Supplementary figures and images for: Marine microbial communities of the Great Barrier Reef lagoon are influenced by riverine floodwaters and seasonal weather events
Source: PeerJ. 2016 Jan 5;4:e1511. doi: 10.7717/peerj.1511 (PMC4734448; doi:10.7717/peerj.1511)

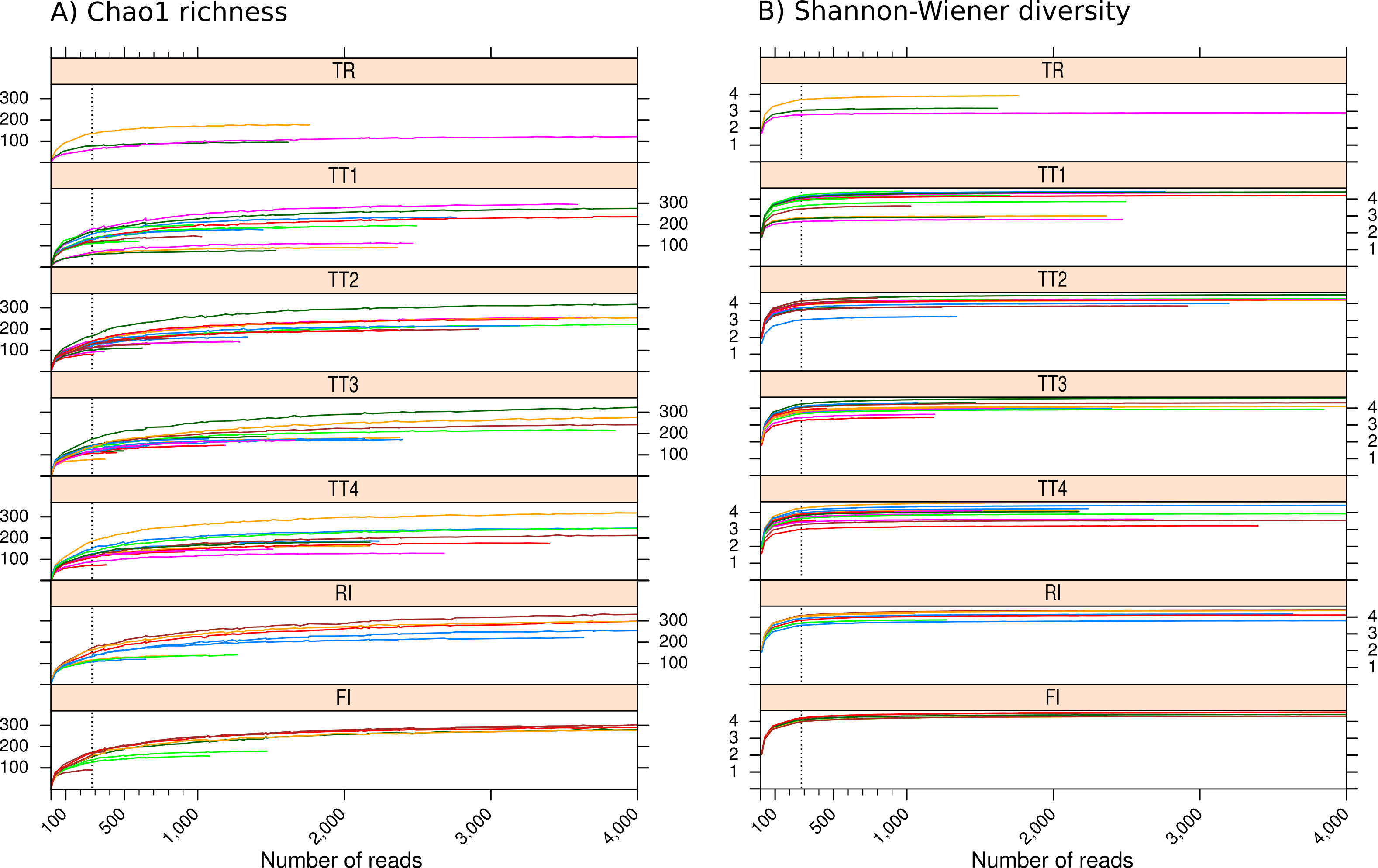

Supplement: Figure S1 — Rarefaction curves of the bacterial and archaeal communities based on 100 bootstrap repetitions and showing (A) the Chao1 richness and (B) the Shannon-Wiener diversity index. The dashed line indicates the rarefaction depth of 279 used for the rest of this study. [file peerj-04-1511-s001.png]

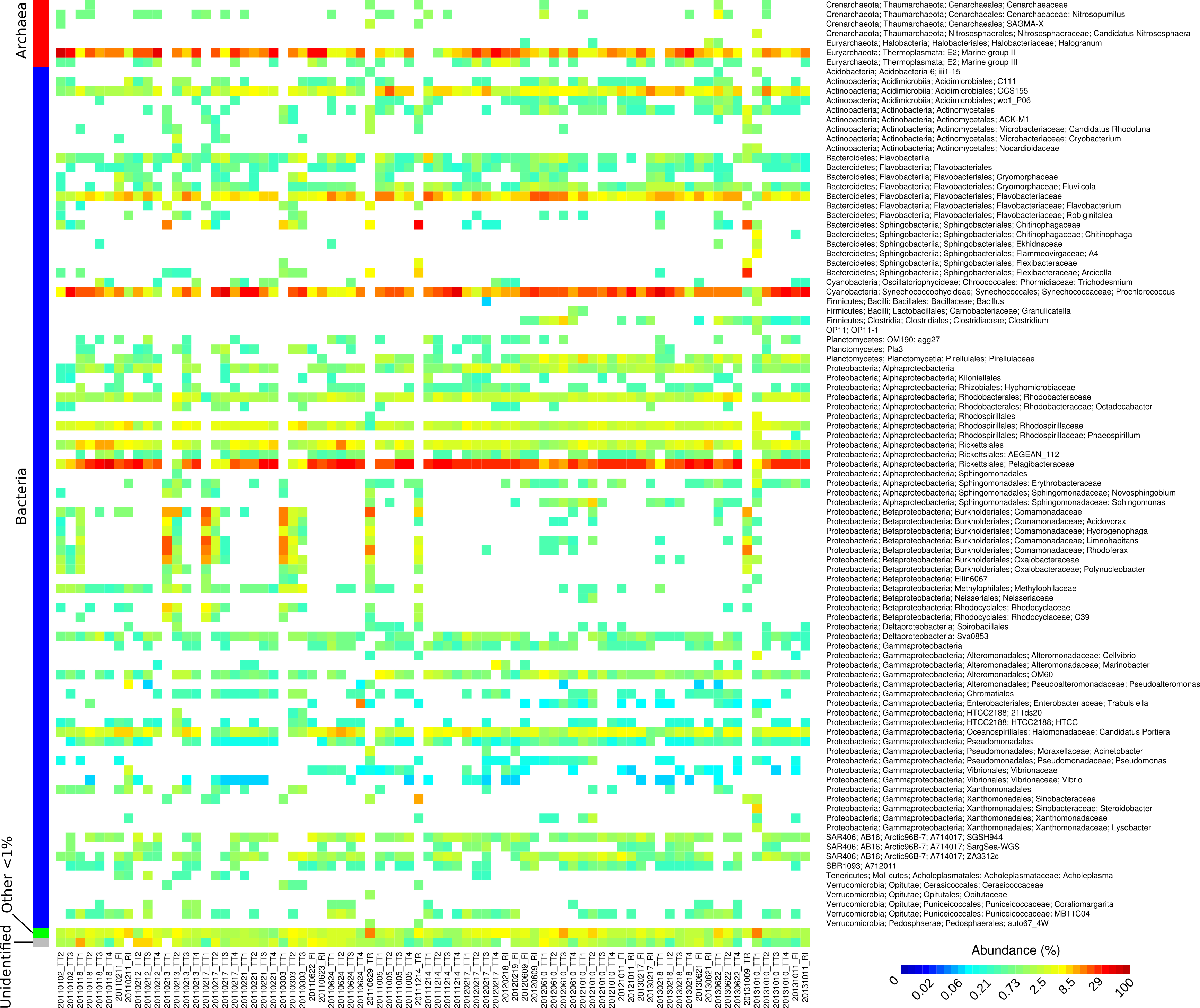

Supplement: Figure S2 — Heatmap showing the estimated relative abundance of microbial genera (archaeal and bacterial) in this survey. Sample names are indicated at the bottom by their date and location, conforming to the specification YYYYMMDD˙site. [file peerj-04-1511-s002.png]

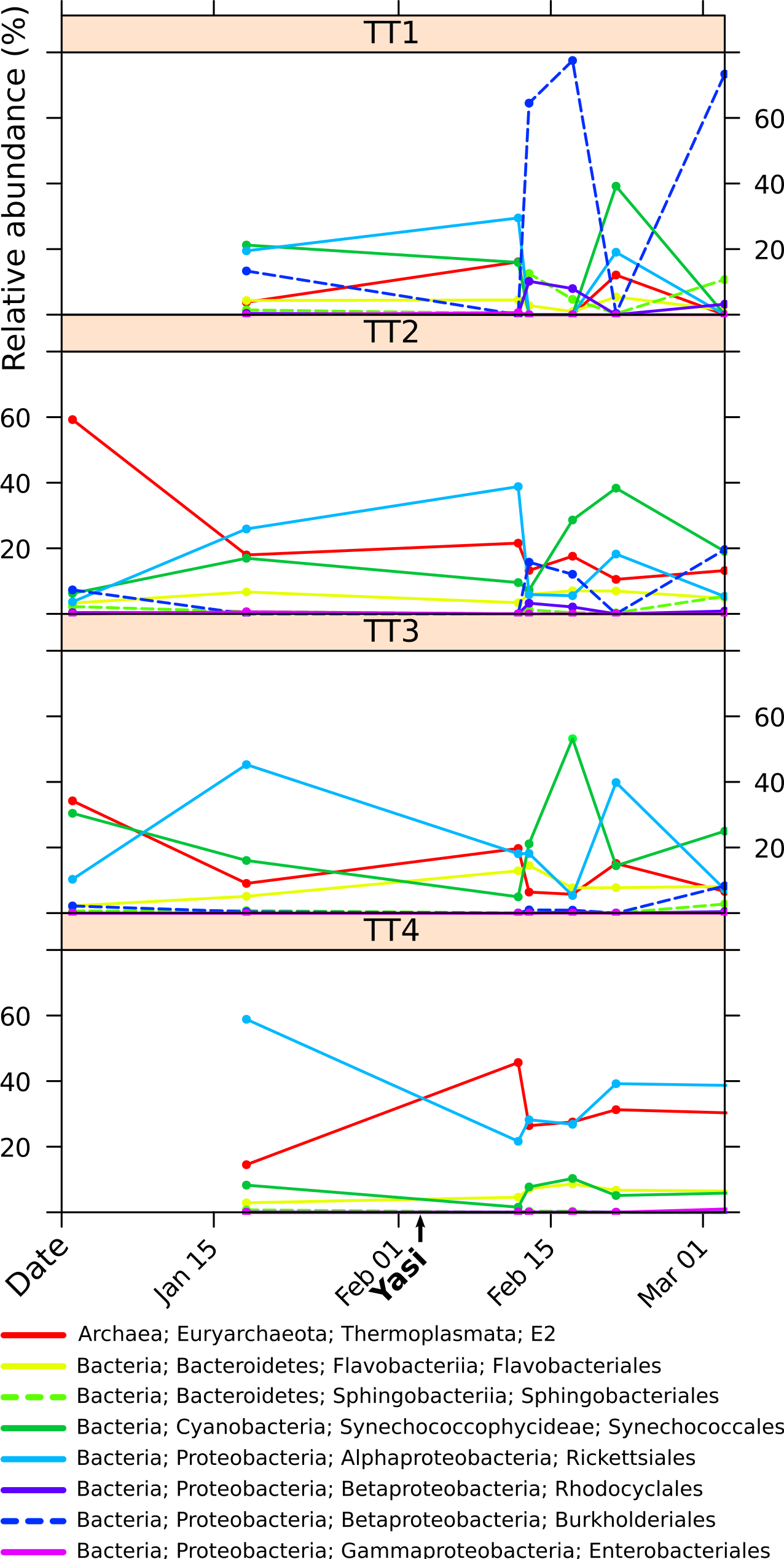

Supplement: Figure S3 — Dynamics of microbial communities (Bacteria and Archaea) in the Tully transect during the 2011 wet season. These microbial profiles were rarefied to 165 counts instead of 279 to allow the inclusion of sample 20110212˙TT1. Dashed lines indicates taxa characteristic of river samples. [file peerj-04-1511-s003.png]

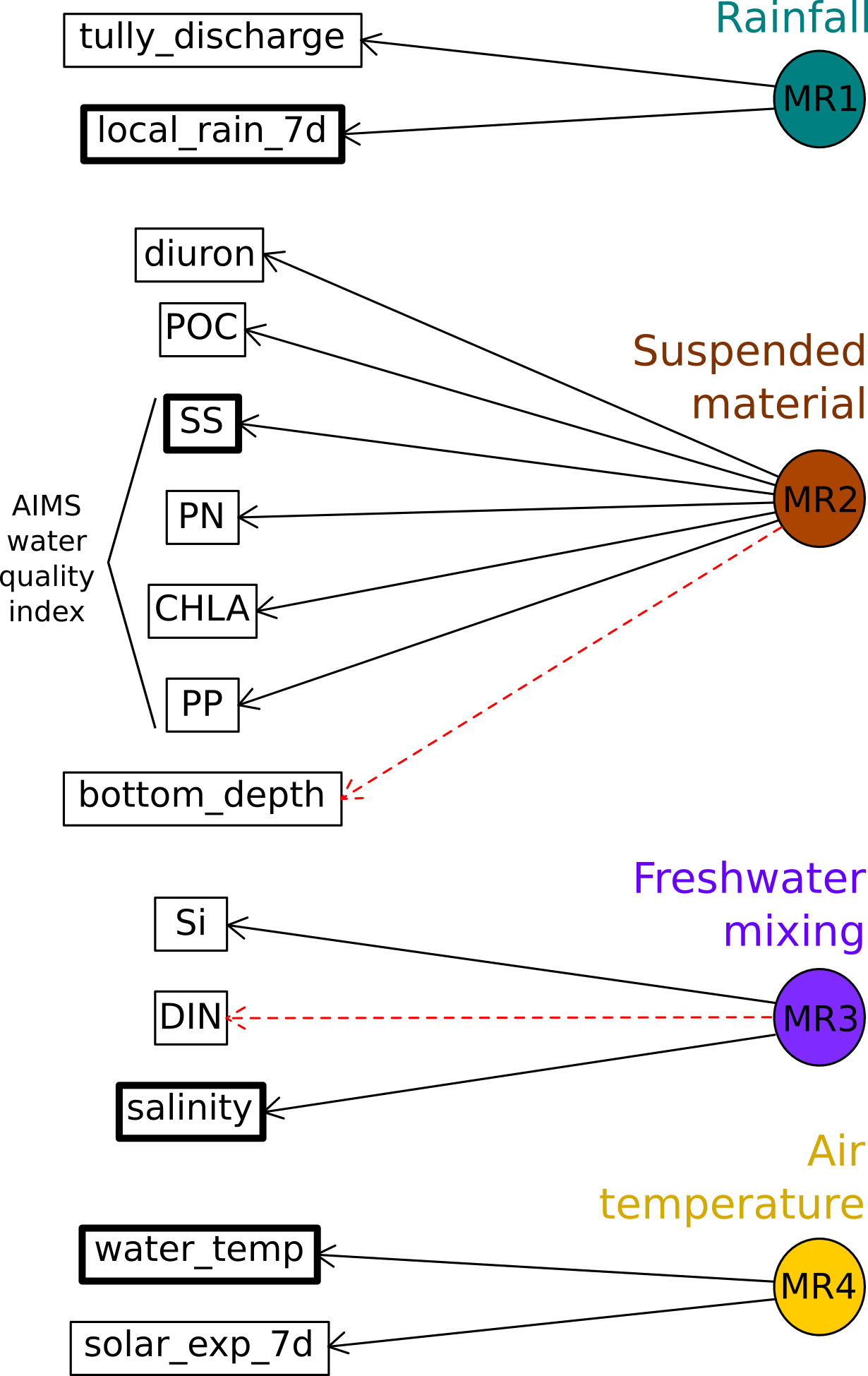

Supplement: Figure S4 — Solid and dashed arrows depict positive and negative relationships, respectively. Environmental parameters are Tully river discharge (tully˙discharge), rainfall in the last 7 days (rain˙7d), amount of diuron (diuron), particulate organic carbon (POC), suspended solids (SS), particulate nitrogen (PN), chlorophyll a (CHLA), particulate phosphorus (PP), bottom depth (bottom˙depth), silica (Si), dissolved inorganic nitrogen (DIN), salinity, water temperature (water˙temp) and solar exposure in the last 7 days (solar˙exp˙7d). The representative parameter chosen for each factor is in bold. [file peerj-04-1511-s004.png]

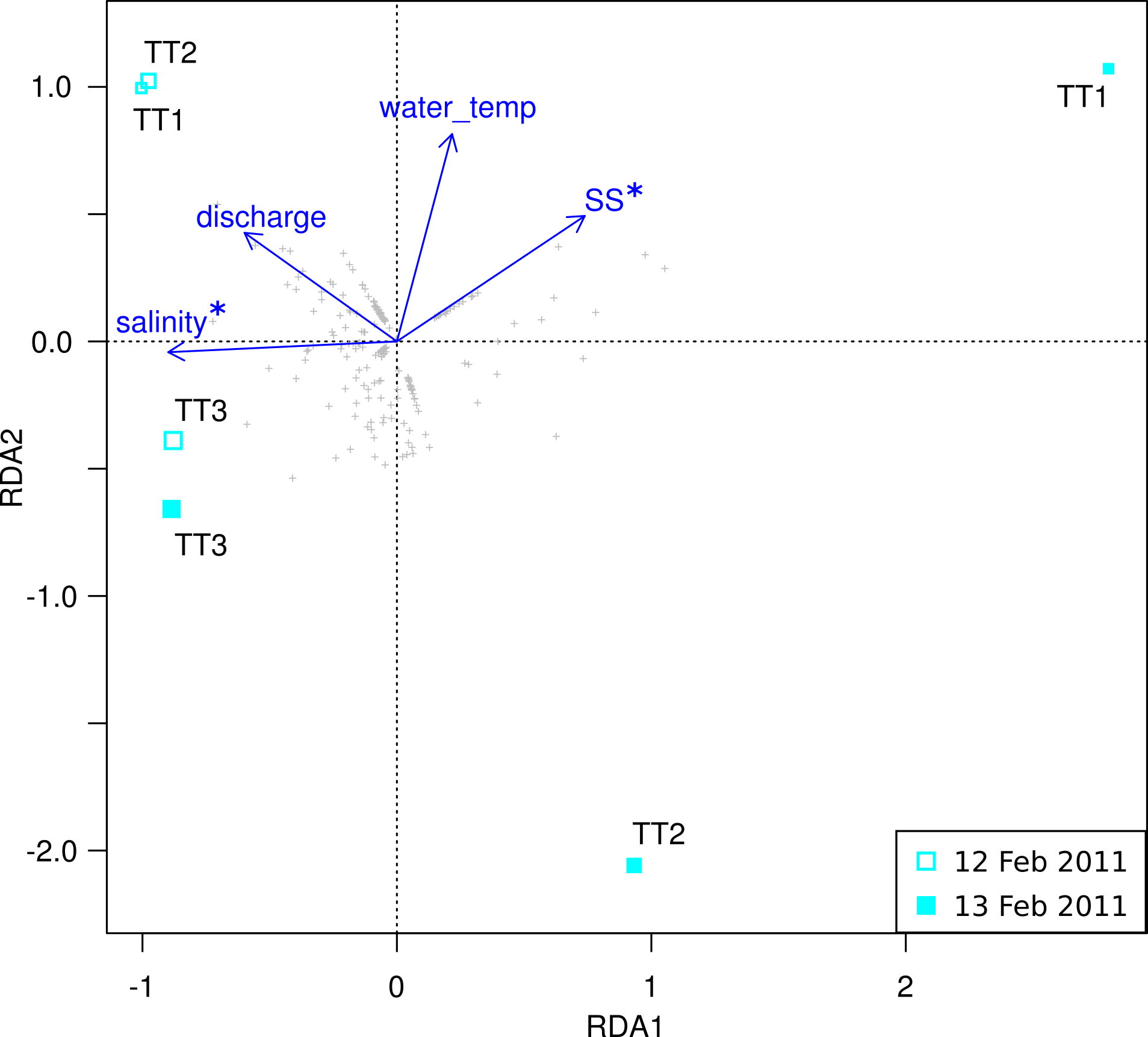

Supplement: Figure S5 [file peerj-04-1511-s007.png]

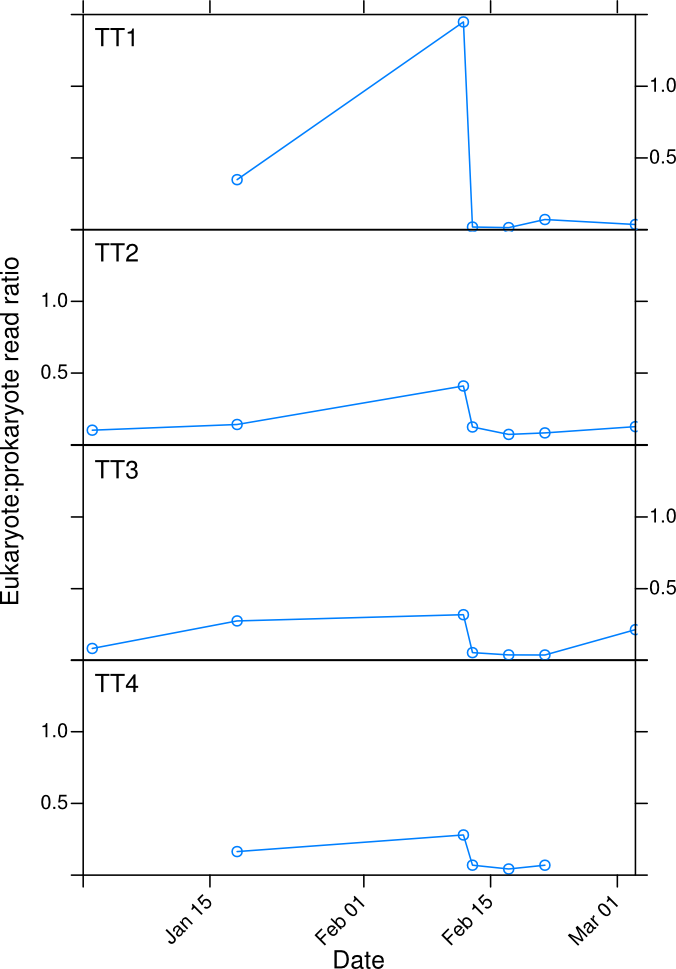

Supplement: Figure S6 — Changes in the ratio of number of eukaryotic to prokaryotic reads during the wet season 2011 (January–March) near the Tully River mouth. [file peerj-04-1511-s009.png]
