# Supplementary material for: Marine microbial communities of the Great Barrier Reef lagoon are influenced by riverine floodwaters and seasonal weather events
Source: PeerJ. 2016 Jan 5;4:e1511. doi: 10.7717/peerj.1511 (PMC4734448; doi:10.7717/peerj.1511)
Supplement: Supplementary file 2 [file peerj-04-1511-s005.odt]

| Sampling site | Nearest influencing river | Distance to river mouth (km) | Classification |
| --- | --- | --- | --- |
| TT1 | Tully River | 2.01 | Plume |
| TT2 | Tully River | 8.75 | Plume |
| TT3 | Tully River | 14.8 | Plume |
| TT4 | Tully River | 30.8 | Marine |
| RI | Johnstone River | 31.5 | Marine |
| FI | Russel River | 34.1 | Marine |
