# Supplementary material for: Marine microbial communities of the Great Barrier Reef lagoon are influenced by riverine floodwaters and seasonal weather events
Source: PeerJ. 2016 Jan 5;4:e1511. doi: 10.7717/peerj.1511 (PMC4734448; doi:10.7717/peerj.1511)
Supplement: Supplementary file 3 [file peerj-04-1511-s006.odt]

| Years | 2010–11 | 2011–12 | 2012–13 | 2013–14 |
| --- | --- | --- | --- | --- |
| Total solar exposure (MJ/m²) | 6,480 | 6,999 | 7,439 | 6,845 |
| Average water temperature (C) | 24.6 | 25.5 | 24.1 | 24.5 |
| Total rainfall (mm) | 4,803 | 3,351 | 2,337 | 3,293 |
| Total river discharge (GL) | 6,151 | 3,606 | 2,847 | 3,736 |
