# Supplementary material for: Marine microbial communities of the Great Barrier Reef lagoon are influenced by riverine floodwaters and seasonal weather events
Source: PeerJ. 2016 Jan 5;4:e1511. doi: 10.7717/peerj.1511 (PMC4734448; doi:10.7717/peerj.1511)
Supplement: Supplementary file 4 [file peerj-04-1511-s008.odt]

| Season | Greengenes OTU ID | Taxon | Stat | p-value |
| --- | --- | --- | --- | --- |
| Dry | 3597424 | Marine group II | 0.990 | 0.000999 *** |
| 325549 | Pelagibacteraceae | 0.924 | 0.000999 *** |
| 318340 | Rhodobacteraceae | 0.904 | 0.000999 *** |
| 2389403 | Rhodobacteraceae | 0.844 | 0.000999 *** |
| 608710 | Psychrobacter | 0.836 | 0.000999 *** |
| 231054 | Portiera | 0.832 | 0.001998 ** |
| 825917 | Pelagibacteraceae | 0.821 | 0.000999 *** |
| 540364 | Portiera | 0.800 | 0.003996 ** |
| 233621 | SGSH944 | 0.798 | 0.004995 ** |
| 1122818 | Pirellulaceae | 0.784 | 0.010989 * |
| 234135 | Pelagibacteraceae | 0.758 | 0.007992 ** |
| 2847578 | Erythrobacteraceae | 0.727 | 0.022977 * |
| 534328 | Flavobacteriaceae | 0.713 | 0.007992 ** |
| 1147758 | Actinomycetales | 0.680 | 0.023976 * |
| 242181 | Flavobacteriia | 0.661 | 0.009990 ** |
| 1147920 | Pirellulaceae | 0.661 | 0.007992 ** |
| 179922 | Flavobacteriia | 0.659 | 0.018981 * |
| 613851 | Clostridium | 0.635 | 0.048951 * |
| 148521 | Maribacter | 0.623 | 0.023976 * |
| 843122 | Trabulsiella | 0.610 | 0.041958 * |
| 688231 | Marine group II | 0.590 | 0.046953 * |
| - | unidentified 417 | 0.559 | 0.036963 * |
| - | unidentified 100 | 0.559 | 0.047952 * |
| Wet | 4247814 | Rickettsiales | 0.849 | 0.00500 ** |
| 357826 | Hyphomicrobiaceae | 0.800 | 0.00200 ** |
| 104828 | SargSea-WGS | 0.702 | 0.00500 ** |
| 831039 | SGSH944 | 0.669 | 0.00599 ** |
| 262657 | Balneolaceae | 0.666 | 0.01399 * |
| 230524 | Vibrio | 0.640 | 0.01898 * |
| 535304 | Rhodospirillaceae | 0.598 | 0.01399 * |
| 1116418 | Trichodesmium | 0.598 | 0.01199 * |
| 216695 | HTCC2188 | 0.598 | 0.01698 * |
| 326890 | Oceanospirillales | 0.535 | 0.03197 * |
| 559024 | Octadecabacter | 0.535 | 0.03596 * |
